# The difference between Asian and Western in the effect of LDL-C lowering therapy on coronary atherosclerotic plaque: a meta-analysis report

**DOI:** 10.1186/1471-2261-15-6

**Published:** 2015-02-14

**Authors:** Yu-Feng Li, Quan-Zhou Feng, Wen-Qian Gao, Xiu-Jing Zhang, Ya Huang, Yun-Dai Chen

**Affiliations:** The Department of Cardiology, Chinese PLA General Hospital, Fuxing Road 28, Beijing, 100853 China; The First Department of Geriatric Cardiology, Chinese PLA General Hospital, Beijing, 100853 China; The First Clinics, Administrative and Supportive Bureau, Chinese PLA General Logistics Department, Jia 14, Fuxing Road 22, Beijing, 100842 China

**Keywords:** Low-density lipoprotein-cholesterol, Coronary atherosclerotic plaque, Intravascular ultrasound, Coronary artery disease, Western, Asian

## Abstract

**Background:**

The different effects of LDL-C levels and statins therapy on coronary atherosclerotic plaque between Western and Asian remain to be settled.

**Methods:**

PubMed, EMBASE, and Cochrane databases were searched from Jan. 2000 to Sep. 2014 for randomized controlled or blinded end-points trials assessing the effects of LDL-C lowering therapy on regression of coronary atherosclerotic plaque (CAP) in patients with coronary heart disease by intravascular ultrasound. The significance of plaques regression was assessed by computing standardized mean difference (SMD) of the volume of CAP between the baseline and follow-up.

**Results:**

Twenty trials (ten in the West and ten in Asia) were identified. For Westerns, Mean lowering LDL-C by 49.4% and/or to level 61.9 mg/dL in the group of patients with baseline mean LDL-C 123.2 mg/dL could significantly reduce the volume of CAP at follow up (SMD −0.156 mm^3^, 95% CI −0.248 ~ −0.064, *p* = 0.001). LDL-C lowering by rosuvastatin (mean 40 mg daily) could significantly decrease the volumes of CAP at follow up. For Asians, Mean lowering LDL-C by 36.1% and/or to level 84.0 mg/dL with baseline mean LDL-C 134.2 mg/dL could significantly reduce the volume of CAP at follow up (SMD −0.211 mm^3^, 95% CI −0.331 ~ −0.092, *p* = 0.001). LDL-C lowering by rosuvastatin (mean 14.1 mg daily) and atorvastatin (mean 18.9 mg daily) could significantly decrease the volumes of CAP at follow up.

**Conclusions:**

There was a different effect of LDL-C lowering on CAP between Westerns and Asians. For regressing CAP, Asians need lower dosage of statins or lower intensity LDL-C lowering therapy than Westerns.

## Background

Atherosclerotic plaque is the hallmark and cornerstone of atherosclerotic disease. Disruption of coronary atherosclerotic plaque (CAP) may lead to sudden cardiac death, acute myocardial infarction, or unstable angina [1]. Intravascular ultrasound (IVUS) is considered to be gold standard for measurement of atherosclerotic plaque [2].

The meta-analysis of twenty trials evaluated the effects of LDL-C lowering on CAP indicated that intensive LDL-C lowering with statins could slow atherosclerotic plaque progression and lead to plaque regression [3]. But the meta-analysis did not investigate the effects of LDL-C lowering on CAP in different race.

In this meta-analysis, we investigated the difference between Western and Asian in the effect of LDL-C lowering therapy on the progression of the CAP from the current trials on LDL-C lowering therapy retarding the progression of the CAP and identified the different targets of LDL-C that result in the regression of the CAP for Western and Asian.

## Methods

Materials and methods of this meta-analysis were detailed in the paper by Gao et al. [3].

### Search strategy and selection criteria

An electronic literature search was performed to identify all relevant studies published in PubMed, EMBASE, and Cochrane databases in the English language from Jan. 1, 2000 to Sep. 13, 2014, using the terms “atherosclerosis” and “cholesterol blood level”. Trials were included using the criteria as: 1) randomized controlled or prospective, blinded end-points trials, and its primary end point was CAP change detected by IVUS; 2) report of LDL-C levels at baseline and follow-up; 3) data on the volume of CAP at baseline and follow-up, and volume of CAP was calculated as vessel volume minus lumen volume; Exclusion criteria were: 1) only CAP area or volume index or percent atheroma volume were detected; 2) the levels of LDL-C at baseline or follow-up were not provided; and 3) target plaques were unstable.

### Data extraction and quality assessment

Two investigators independently reviewed all potentially eligible studies and collected data on patient and study characteristics, and any disagreement was resolved by consensus. The primary end point of this study was the volume change of CAP detected by IVUS. Quality assessments of trials were evaluated with Jadad quality scale.

### Data synthesis and analysis

Volume changes of CAP from baseline to follow-up were analyzed using standardized mean differences (SMD).

Volume changes of plaque in every arm were used for pooled analysis. The trials were firstly grouped into group Western and Asian according to the location of the trials. Then, according to the levels and the reducing percentage of LDL-C at follow-up, the arms were grouped to following groups: ≤70, >70 ≤ 100 HP, >70 ≤ 100 MP, >70 ≤ 100 LP, >100 mg/dL; and <0, ≥0 < 30, ≥30 < 40, ≥40 < 50, ≥50% respectively [3], to investigate the effect of different levels of LDL-C at follow up on CAPs. According to statins, the arms were grouped to: rosuvastatin, atorvastatin, pitavastatin, simvastatin, fluvastatin and pravastatin groups, to investigate the effect of different statins on CAPs. The volume of CAP at follow up was compared with that at baseline to evaluate effect of LDL-C levels on regression of CAP.

Heterogeneity across trials (arms) was assessed via a standard χ^*2*^ test with significance being set at *p* < 0.10 and also assessed by means of *I*^*2*^ statistic with significance being set at *I*^*2*^ > 50%. Pooled analyses were calculated using fixed-effect models, whereas random-effect models were applied in case of significant heterogeneity across trials (arms). Sensitivity analyses (exclusion of one study at one time) were performed to determine the stability of the results. Publication bias was assessed using the Egger regression asymmetry test. Statistical analyses were performed using STATA software 12.0 (StataCorp, College Station, Texas).

All continuous variables were expressed as mean ± SD, and continuous variables were compared between the Western and Asian groups using Student’s t test (SigmaStat 3.5). A P value <0.05 was considered to be statistically significant.

## Results

### Eligible studies

**Figure 1:**
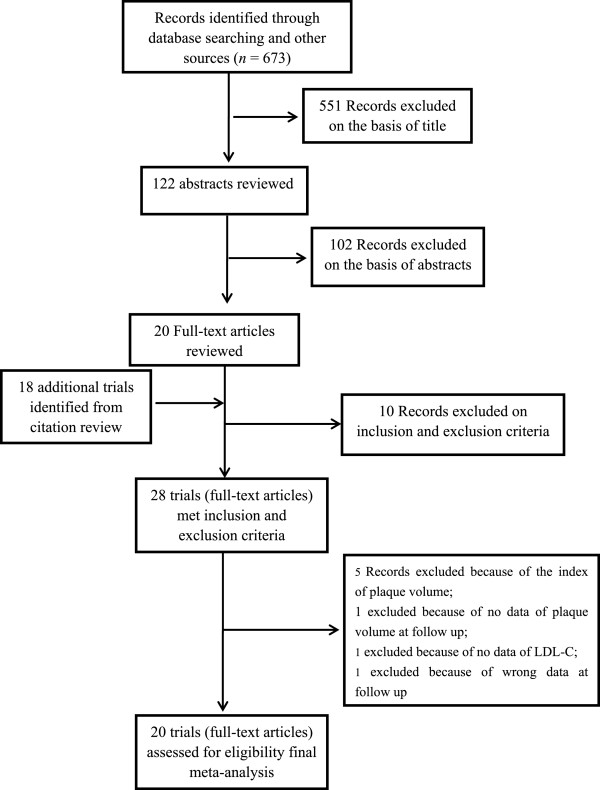
**Flow diagram of study-screening process.**

The flow of selecting studies for the meta-analysis was shown in Figure 1. Briefly, of the initial 673 articles, one hundred and twenty-two of abstracts were reviewed, resulting in exclusion of 102 articles, and 20 articles were reviewed in full text, resulting in exclusion of 10 trials and inclusion of 18 additional trials cited in the 20 articles. Twenty two RCTs [4–25] and six blinded end-points trial [26–31] were carefully evaluated, and eight trials [4, 8, 9, 18, 19, 21, 27, 31] were excluded because of specific the index of plaque or lack of some data. Sixteen RCT (ESTABLISH [11], REVERSAL [10], A-PLUS [5], ACTIVATE [6], ILLUSTRATE [7], JAPAN-ACS [20], REACH [14], SATURN [16], ARTMAP [17], ERASE [23], STRADIVARIUS [24], PERISCOPE [25], and trials by Yokoyama M [12], by Kawasaki M [13], by Hong MK [15], and Tani S [22]) and four blinded end-points trial (ASTEROID [26], COSMOS [29], trial by Jensen LO [28] and trial by Nasu K [30]) were finally analyzed.

**Figure 2:**
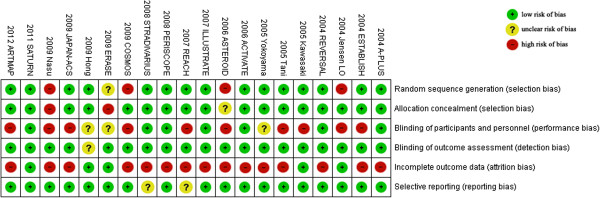
**Methodological quality summary of each included trial.**

**Figure 3:**
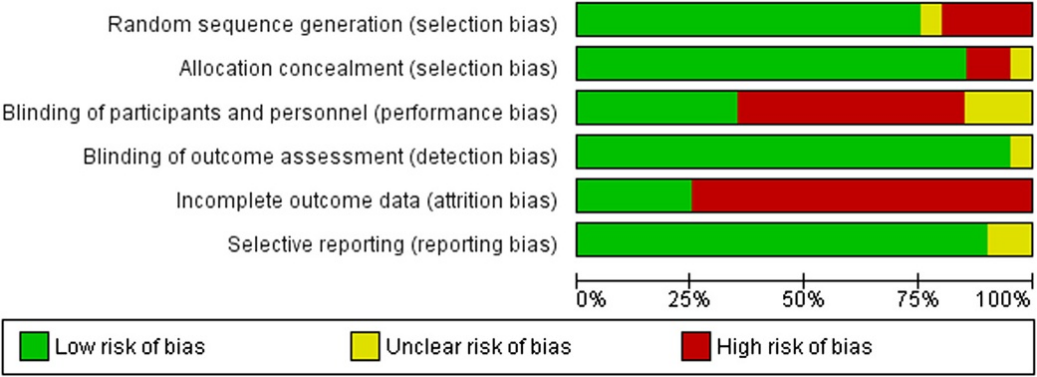
**Methodological quality graph: each methodological quality item presented as percentages across all included studies.**

The characteristics of the included trials were as same as in the study [3] and shown in Table 1. Briefly, among the 20 trials, 10 trials are completed in European, America and Australia [10, 5–7, 16, 23–26, 28], 10 in Asia [20, 11–15, 17, 22, 29, 30], and there were 15 trials assessing statins (statin vs. usual care in 6 trials [11–14, 22, 30]; intensive statin vs. moderate statin treatment in 5 trials [10, 15–17, 20]; follow up vs baseline in 3 trial [26, 28, 29], before acute coronary syndrome (ACS) vs after ACS in one trial [23]), 2 trials assessing enzyme acyl–coenzyme A: cholesterol acyltransferase (ACAT) inhibition [5, 6], one trial assessing cholesteryl ester transfer protein (CETP) inhibitor torcetrapib [7], one trial assessing a decreasing obesity drug: rimonabant [24], and one trial assessing glucose-lowering agents [25]. Overall, 5910 patients with coronary heart disease (CHD) underwent serial IVUS examination for evaluating regression of CAP. Follow-up periods ranged from 2 to 24 months. The levels of LDL-C of each arm at baseline and follow-up were shown in Table 2.Risk of bias of included studies, evaluated through Cochrane’s methods, showed an overall acceptable quality of selected trials (Figures 2 and 3).

**Table 1:** **Features of participating trials**

Authors and trial name	Trial type and location	Objective	Year	N T/C	Study population	LDL-C at follow up	LDL-C reducing percentage	Treatments	Follow up	Main Results or Conclusion
Okazaki S^11^; ESTABLISH	RCT: prospective, open-label, randomized, single center study. Japan	Effects of statins on changes in plaque by IVUS	2004	24/24	ACS	70/119	−44/-0.004	Ato 20 vs Diet	6	Plaque volume was sigificantly reduced in the Ato group compared with the control group.
Nissen SE^10^; REVERSAL	RCT: Double-blind, randomized active control multicenter trial; USA	Effects of statins (intensive or moderate) on changes in plaque by IVUS	2004	253/249	CAD	79/110	−46/-25	Ato 80 vs Pra40	18	Ato reduced progression of coronary plaque compared with Pra. Compared with baseline values, Ato had no change in atheroma burden, whereas patients treated with Pra showed progression of coronary plaque.
Tardif JC^5^; A-PLUS	RCT: international, multicenter, double-blind, placebo-controlled, randomized trial. Canada, USA	Effects of different dosage of avasimibe on changes in plaque by IVUS	2004	108/98/117/109	CAD	100/102/101/91	7.8/9.1/10.9/1.7	Ava50, 250, and 750 vs Placebo on the basis of LDL-C < 125	18	Avasimibe did not favorably alter coronary atherosclerosis as assessed by IVUS.
Jensen LO^28^	Open non placebo controlled serial investigation; blinded end-points. Denmark	To investigate the effect of lipid lowering by simvastatin on coronary atherosclerotic plaque volumes and lumen.	2004	40	CAD	85	−46.3	Sim 40	15	Lipid-lowering therapy with Sim is associated with a significant plaque regression in coronary arteries.
Yokoyama M^12^	RCT: randomized, single center. Japan	Effects of statins on changes in plaque by IVUS	2005	29/30	stabl angina	87/124	−35/-0.075	Ato 10 vs Diet	6	Treatment with Ato may reduce volumes of coronary plaques.
Kawasaki M^13^	RCT: randomization, open-label, single-center study. Japan	Effects of statins on changes in plaque by IVUS	2005	17/18/17	stable angina	95/102/149	−39/-32/-0.02	Ato 20, Pra 20 vs Diet	6	Treatment with Ato and Pra may not significantly reduce volumes of coronary plaques.
Tani S^22^	RCT: a prospective, single-center, randomized, open trial. Japan	Investigated the effects of pravastatin on the serum levels of MDA-LDL and coronary atherosclerosis.	2005	52/23	stable angina	104/120	−20/-2.4	Pra 10–20 vs con	6	Plaque volume was sigificantly reduced in the Pra group compared with the control group.
Nissen SE^6^; ACTIVATE	RCT: randomized, multicenter. USA	Effects of pactimibe on changes in plaque by IVUS	2006	206/202	CAD	91/86	−9.6/-14.9	Pac100 vs Placebo	18	Pac is not an effective strategy for limiting atherosclerosis and may promote atherogenesis.
Nissen SE^26^; ASTEROID	Prospective, open-label blinded end-points. USA, Germany, France, Canada	Effects of Statins with different levels of LDL-C on changes in plaque by IVUS	2006	349	CAD	61	−53.2	Ros 40	24	Therapy using Ros can result in significant regression of atherosclerosis.
Yamada T^14^; REACH	RCT: open-labeled, randomized, multicenter study. Japan	Evaluate the effect of marked reduction of LDL-C in patients with CHD on progression of atherosclerosis.	2007	26/32	stable angina	83/115	−43/0	Ato 5 vs Con	12	Ato treatment prevented the further progression of atherosclerosis by maintaining LDL-C below 100 mg/dl in patients with CHD.
Nissen SE^7^; ILLUSTRATE	RCT: prospective, randomized, multicenter, double-blind clinical trial. North America or Europe	Effects of CETP inhibitor on changes in plaque by IVUS	2007	446/464	CAD	87/70	6.6/-13.3	Ato10-80 vs Ato + Tor 60 on the basis of LDL-C ≤ 100 by Ato	24	The Tor was associated with a substantial increase in HDL-C and decrease in LDL –C, and there was no significant decrease in the progression of coronary atherosclerosis.
Nissen SE^25^; PERISCOPE	RCT: prospective, randomized, multicenter, double-blind clinical trial. USA	To compare the effects of pioglitazone, and glimepiride on the progression of coronary atherosclerosis in patients with type 2 diabete and CAD	2008	181/179	CAD,DM	96.1/95.6	1.8/2.2	Gli1-4 mg vs Pio 15-45 mg on bases of statins therapy	18	In patients with type 2 diabetes and CAD, treatment with Pio resulted in a significantly lower rate of progression of coronary atherosclerosis compared with Gli.
Nissen SE^24^; STRADIVARIUS	RCT: Randomized, double-blinded, placebo -controlled, 2-group, parallel-group trial. North America, Europe, and Australia	The effect of rimonabant on regression of coronary disease in patients with the metabolic syndrome and CAD	2008	335/341	CAD,Obesity	87.6/86.3	−4.7/-3.6	Rim 20 mg vs Placebo on bases of statins therapy	18	Rim can reduce progression of coronary plaque, and increase HDL-C levels, decrease triglyceride levels.
Hiro T^20^; JAPAN-ACS	RCT: prospective, randomized, open-label, parallel group, multicenter. Japan	Effects of statins on changes in plaque by IVUS	2009	127/125	ACS	84/81	−36/-36	Ato 20 vs Pit 4	10	The administration of Pit or Ato in patients with ACS equivalently resulted in significant regression of coronary plaque volume.
Takayama T; COSMOS^29^	Prospective, open-label blinded end-points multicenter trial. Japan	Evaluate the effect of rosuvastatin on plaque volume in patients with stable CAD, including those receiving prior lipid-lowering therapy	2009	126	stable angina	83	−38.6	Ros <20	14	Ros exerted significant regression of coronary plaque volume in Japanese patients with stable CAD.
Rodés-Cabau; ERASE^23^	RCT: multicenter randomized placebo-controlled. Canada	Evaluate the early effects of newly initiated statin therapy on coronary atherosclerosis as evaluated by IVUS.	2009	38/36	ACS	77/63	8.5/-37	Before ACS vs After ACS	<2	Newly initiated statin therapy is associated with rapid regression of coronary atherosclerosis.
Nasu K^30^	Prospective and multicenter study with nonrandomized and non-blinded design, but blinded end. Japan	Evaluate the effect of treatment with statins on the progression of coronary atherosclerotic plaques of a nonculprit vessel by serial IVUS.	2009	40/39	stable angina	98.1/121	−32.3/-1.1	Flu 60 vs Con	12	One-year lipid-lowering therapy by Flu showed significant regression of plaque volume.
Hong MK^15^	RCT: randomized control trial. Korea.	Evaluated the effects of statin treatments for each component of coronary plaques.	2009	50/50	stable angina	78/64	−34.5/-44.8	Sim 20 vs Ros 10	12	Statin treatments might be associated with significant changes in necrotic core and fibrofatty plaque volume.
Nicholls SJ; SATURN^16^	RCT: a prospective, randomized, multicenter, double-blind clinical trial. USA	Compare the effect of these two intensive statin regimens on the progression of coronary atherosclerosis.	2011	519/520	CHD	70.2/62.6	−41.5/-47.8	Ato 80 vs Ros 40	24	Maximal doses of Ros and Ato resulted in significant regression of coronary atherosclerosis.
Lee CW^17^; ARTMAP	RCT: a prospective, single-center, open-label, randomized comparison trial. Korea.	Compared the effects of atorvastatin 20 mg/day versus rosuvastatin 10 mg/day on mild coronary atherosclerotic plaques.	2012	143/128	stable angina	56/53	−47/-49	Ato 20 vs Ros 10	6	Usual doses of Ato and Ros induced significant regression of coronary atherosclerosis in statin-naive patients.

*Abbreviations*: *T* Treatment, *C* Control, *RCT* randomized controlled trials, *IVUS* Intravascular ultrasound, *CAD* Coronary artery disease, *ACS* Acute coronary syndrome, *CHD* Coronary heart disease, *Ato* Atorvastatin, *Ros* Rosuvastatin, *Pra* Pravastatin, *Pit* Pitavastatin, *Sim* Simvastatin, *Flu* Fluvastatin, *Con* Control, *Pac* Pactimibe, *Tor* Torcetrapib, Ava 50, 250, 750, Avasimibe 50, 250, 750 mg, *T/C* Treat/Control, *Gli* Glimepiride, *Pio* Pioglitazone, *Rim* Rimonabant.

**Table 2:** **The levels of LDL-C at baseline and follow up in each arm of included trials**

Authors	Trial name	Management in each arm	N	LDL-C level	
				At baseline	At follow-up
Tardif JC	A-PLUS	Avasimibe50	108	92.8 ± 1.7	100*
Tardif JC	A-PLUS	Avasimibe250	98	93.4 ± 1.6	101.9*
Tardif JC	A-PLUS	Avasimibe750	117	91.4 ± 1.6	101.4*
Tardif JC	A-PLUS	Placebo	109	89.6 ± 1.6	91.1*
Okazaki S	ESTABLISH	Control	24	123.9 ± 35.3	119.4 ± 24.6
Okazaki S	ESTABLISH	Atorvastatin	24	124.6 ± 34.5	70.0 ± 25.0
Yokoyama M		Control	30	131.5 ± 23#	124.5 ± 24.1#
Yokoyama M		Atorvastatin	29	133 ± 13	87 ± 29
Nissen SE	REVERSAL	Atorvastatin	253	150.2 ± 27.9	78.9 ± 30.2
Nissen SE	REVERSAL	Pravastatin	249	150.2 ± 25.9	110.4 ± 25.8
Nissen SE	ACTIVATE	Pactimibe	206	101.4 ± 27.7	91.3
Nissen SE	ACTIVATE	Placebo	202	101.5 ± 31.1	86.4
Nissen SE	ILLUSTRATE	Atorvastatin	446	84.3 ± 18.9	87.2 ± 22.6
Nissen SE	ILLUSTRATE	Atorva + torcetrapib	464	83.1 ± 19.7	70.1 ± 25.4
Kawasaki M		Control	17	152 ± 20	149 ± 24
Kawasaki M		Pravastatin	18	149 ± 19	102 ± 13
Kawasaki M		Atorvastatin	17	155 ± 22	95 ± 15
Hiro T	JAPAN-ACS	Pitavastatin	125	130.9 ± 33.3	81.1 ± 23.4
Hiro T	JAPAN-ACS	Atorvastatin	127	133.8 ± 31.4	84.1 ± 27.4
Nissen SE	ASTEROID	Rosuvastatin	349	130.4 ± 34.3	60.8 ± 20.0
Takayama T	COSMOS	Rosuvastatin	126	140.2 ± 31.5	82.9 ± 18.7
Lee CW	ARTMAP	Atorvastatin	143	110 ± 31	56 ± 18
Lee CW	ARTMAP	Rosuvastatin	128	109 ± 31	53 ± 18
Yamada T	REACH	Atorvastatin	26	123 ± 17	83 ± 22
Yamada T	REACH	Control	32	115 ± 14	115 ± 30
Nasu K		Fluvastatin	40	144.9 ± 31.5	98.1 ± 12.7
Nasu K		Control	39	122.3 ± 18.9	121.0 ± 21.2
Nicholls SJ	SATURN	Atorvastatin	519	119.9 ± 28.9	70.2 ± 1.0
Nicholls SJ	SATURN	Rosuvastatin	520	120.0 ± 27.3	62.6 ± 1.0
Hong MK		Simvastatin	50	119 ± 30	78 ± 20
Hong MK		Rosuvastatin	50	116 ± 28	64 ± 21
Tani S		Pravastatin	52	130 ± 38	104 ± 20
Tani S		Control	23	123 ± 28	120 ± 30
Rodés-C Bef	ERASE	Statins before ACS	38	71 ± 23	77 ± 25
Rodés-C Aft	ERASE	Statins after ACS	36	100 ± 30	63 ± 17
Jensen LO		Simvastatin	40	158.7 ± 30.6	85.1 ± 22.1
Nissen SE	PERISCOPE	Statins + Gli	181	94.4 ± 32.9	96.1 ± 30.4
Nissen SE	PERISCOPE	Statins + Pio	179	93.5 ± 30.7	95.6 ± 28.9
Nissen SE	STRADIVARIUS	Statins + Rim	335	91.9 ± 27.9	87.6 ± 30.5
Nissen SE	STRADIVARIUS	Statins + Con	341	89.5 ± 32.2	86.3 ± 30.3

Note: *calculated on the bases of baseline levels and change percentage at follow up^5^.

^#^calculated according to Figure 2 in the paper^12^.

### The effect of the levels of LDL-C at follow-up on regression of coronary atherosclerotic plaque in Western and Asian

**Figure 4:**
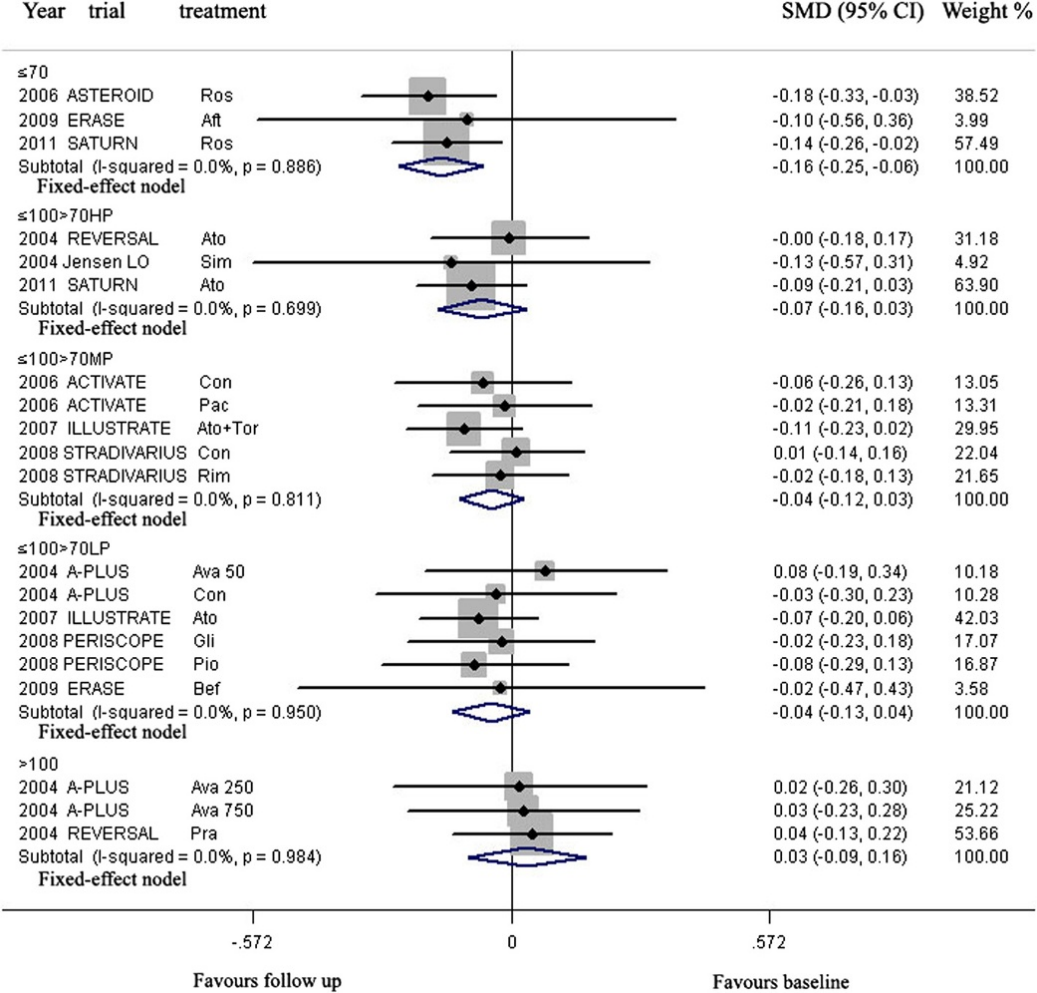
**Meta- analysis of the effects of reduction levels of LDL-C at follow up on the regression of coronary atherosclerotic plaque in Western.** Abbreviations: Ato, Atorvastatin; Ros, Rosuvastatin; Pra, Pravastatin; Pit, Pitavastatin; Sim, Simvastatin; Flu, Fluvastatin; Con, Control; Pac, Pactimibe; Tor, Torcetrapib, Ava 50, 250, 750, Avasimibe 50, 250, 750 mg; Bef, before ACS; Aft, after ACS; Gli, Glimepiride; Pio, Pioglitazone; Rim, Rimonabant.

**For Western**, meta-analysis indicated that LDL-C lowering in group ≤70 mg/dL could lead to regression of CAP, but LDL-C lowering in group >70 ≤ 100 HP, >70 ≤ 100 MP, >70 ≤ 100 LP and >100 mg/dL could not (Figure 4, Table 3).

**Table 3:** **Results of meta-analysis in each group and mean CAP volume in each group at baseline and follow up in Western and Asian**

Group		Included arms (case)	CAP volume at baseline (mm ^3^ )	CAP volume at follow up (mm ^3^ )	Pooled SMD (95% CI, ***p*** )	Heterogeneity test		Sensitivity analyses		Egger’s test
						χ ^***2***^ test ( ***p*** )	***I*** ^***2***^	Lower SMD (95% CI)	Upper SMD (95% CI)	
Western	<70 mg	3(905)	171.4 ± 32.7	160.6 ± 29.7	−0.156(−0.248 ~ −0.064, 0.001)	0.33(0.886)	0	−0.139 (−0.257 ~ −0.021) Without 2006 ASTEROID Ros	−0.175 (−0.317 ~ −0.034) Without 2011 SATURN Ros	0.789
	>70 ≤ 100 HPmg	3(812)	151.9 ± 30.4	147.9 ± 31.9	−0.065(−0.136 ~ 0.032, 0.189)	0.71(0.699)	0			0.987
	>70 ≤ 100 MPmg	5(1548)	195.8 ± 2.3	191.8 ± 4.7	−0.045(−0.115 ~ −0.026, 0.215)	1.59(0.811)	0			0.500
	>70 ≤ 100 LPmg	6(1061)	201.2 ± 15.1	197.3 ± 15.0	−0.045(−0.130 ~ 0.040, 0.301)	1.14(0.950)	0			0.241
	>100 mg	3(464)	197.6 ± 3.5	201.1 ± 1.9	0.034(−0.094 ~ 0.163, 0.601)	0.03(0.984)	0			
	>50%	1(349)	212.2 ± 81.3	197.5 ± 79.1	−0.183(−0.332 ~ −0.035, 0.016)					
	>40 ≤ 50%	4(1332)	148.8 ± 24.0	143.1 ± 25.6	−0.095(−0.171 ~ −0.019, 0.014)	1.64(0.651)	0	−0.065 (−0.163 ~ 0.032) Without 2011 SATURN Ros	−0.116 (−0.201 ~ −0.032) Without 2004 REVERSAL Ato	0.804
	>30 ≤ 40%	1(36)	169.1 ± 77.3	161.5 ± 75.2	−0.099(−0.561 ~ 0.363, 0.675)	0.00(0.000)	0			
	>0 ≤ 30%	6(1797)	195.6 ± 2.1	192.9 ± 5.1	−0.032(−0.098 ~ 0.033, 0.335)	2.45(0.784)	0			
	<0%	8(1276)	201.2 ± 13.8	198.3 ± 13.8	−0.034(−0.111 ~ 0.044, 0.396)	1.55(0.981)	0			0.087
Asian	<70 mg	4(345)	192.2 ± 59.9	179.9 ± 53.0	−0.157(−0.307 ~ −0.008, 0.039)	0.24(0.955)	0	−0.126 (−0.314 ~ 0.063) Without 2012 ARTMAP Ros	−0.187 (−0.383 ~ 0.008) Without 2012 ARTMAP Ato	0.970
	>70 ≤ 100 HPmg	8(540)	96.4 ± 99.3	87.5 ± 92.0	−0.211(−0.331 ~ −0.092, 0.001)	2.68(0.913)	0	−0.177 (−0.314 ~ −0.040) Without 2009 JAPAN-ACS Ato	−0.231(−0.368 ~ −0.094) Without 2009 COSMOS Ros	0.083
	>100 mg	8(235)	133.0 ± 139.6	134.3 ± 143.8	−0.029(−0.210 ~ 0.152, 0.750)	2.14(0.952)	0			
	>40 ≤ 50%	4(345)	192.2 ± 56.9	179.9 ± 53.0	−0.157(−0.307 ~ −0.008, 0.039)	0.33(0.955)	0	−0.126 (−0.314 ~ 0.063) Without 2012 ARTMAP Ros	−0.187 (−0.383 ~ 0.008) Without 2012 ARTMAP Ato	0.970
	>30 ≤ 40%	9(558)	98.6 ± 98.5	90.0 ± 91.6	−0.206(−0.324 ~ −0.088, 0.001)	2.91(0.840)	0	−0.172 (−0.306 ~ −0.038) Without 2009 JAPAN-ACS Ato	−0.223 (−0.357 ~ −0.089) Without 2009 COSMOS Ros	0.004
	>0 ≤ 30%	7(217)	130.2 ± 144.9	131.8 ± 149.4	−0.028(−0.216 ~ 0.161, 0.773)	2.14(0.907)	0			

In group ≤70 mg/dL (including three arms) with mean 23.1 months of follow up, the volumes of CAP (160.6 mm^3^) at follow up were significantly decreased, compared with the volumes (171.4 mm^3^) at baseline [SMD −0.156 mm^3^, 95% CI (confidence interval) -0.248 ~ −0.064, *p* = 0.001]. There was no significant heterogeneity among arms (χ^*2*^ for heterogeneity = 0.33, *p* =0.886, *I*^2^ = 0%).

Sensitivity analyses suggested that LDL-C lowering in group ≤70 mg/dL could lead to regression of CAP with reduction of the CAP volume ranged from −0.139 mm^3^ (SMD, 95% CI: −0.257 ~ −0.021) when the arm of 2006 ASTEROID Ros was omitted to −0.175 mm^3^ (SMD, 95% CI: −0.317 ~ −0.034) when the arm of 2011 SATURN Ros was omitted. No publication bias was found, the values of *p* by Egger’s test was 0.789.

**For Asian**, according to the levels of LDL-C at follow-up, the arms were grouped to three groups: ≤70, >70 ≤ 100 HP and >100 mg/dL.

**Figure 5:**
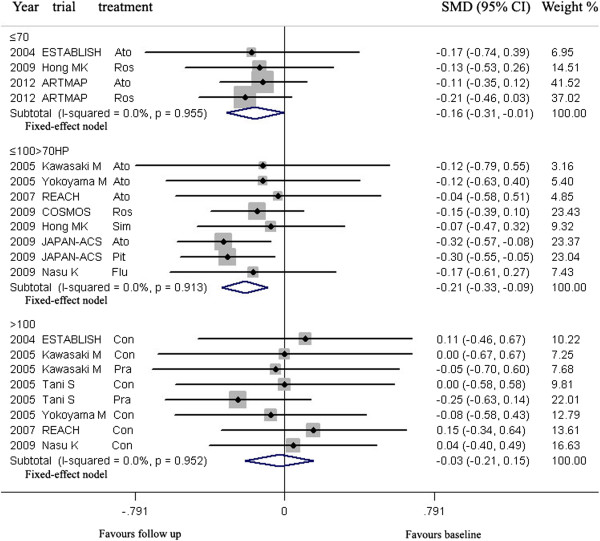
**Meta- analysis of the effects of reduction levels of LDL-C at follow up on the regression of coronary atherosclerotic plaque in Asian.** Abbreviation as in Figure 4.

LDL-C lowering in group ≤70 mg/dL and >70 ≤ 100 HP could lead to regression of CAP, but LDL-C lowering in group >100 mg/dL could not (Figure 5, Table 3).

In group ≤70 mg/dL (including four arms) with mean 6.9 months of follow up and group >70 ≤ 100HP mg/dL (including eight arms) with mean 11.0 months of follow up, the volumes of CAP (179.9, 87.5 mm^3^ respectively) at follow up were significantly decreased, compared with the volumes (192.2, 96.4 mm^3^ respectively) at baseline [SMD −0.157 mm^3^, 95% CI −0.307 ~ −0.008, *p* = 0.039; SMD −0.211 mm^3^, 95% CI −0.331 ~ −0.092, *p* = 0.001; respectively]. There was no significant heterogeneity among arms (χ^*2*^ for heterogeneity = 0.24, *p* =0.955, *I*^2^ = 0% for group ≤70 mg/dL; χ^*2*^ for heterogeneity = 2.68, *p* =0.913, *I*^2^ = 0% for group >70 ≤ 100HP mg/dL).

Sensitivity analyses suggested that LDL-C lowering in group >70 ≤ 100 HP mg/dL could lead to regression of CAP with reduction of the CAP volume ranged from −0.177 mm^3^ (SMD, 95% CI: −0.314 ~ −0.040) when the arm of 2009 JAPAN-ACS Ato was omitted to −0.231 mm^3^ (SMD, 95% CI: −0.368 ~ −0.094) when the arm of 2009 COSMOS Ros was omitted; but that LDL-C lowering in group ≤ 70 mg/dL could not significantly lead to regression of CAP with reduction of the CAP volume when the arm of 2012 ARTMAP Ros or 2012 ARTMAP Ato was omitted (Table 3).

No publication bias was found, the values of *p* by Egger’s test for group ≤70 and >70 ≤ 100HP mg/dL were 0.970, 0.083 respectively.

### The effect of the LDL-C reducing percentage at follow-up on regression of CAP in Western and Asian

**Figure 6:**
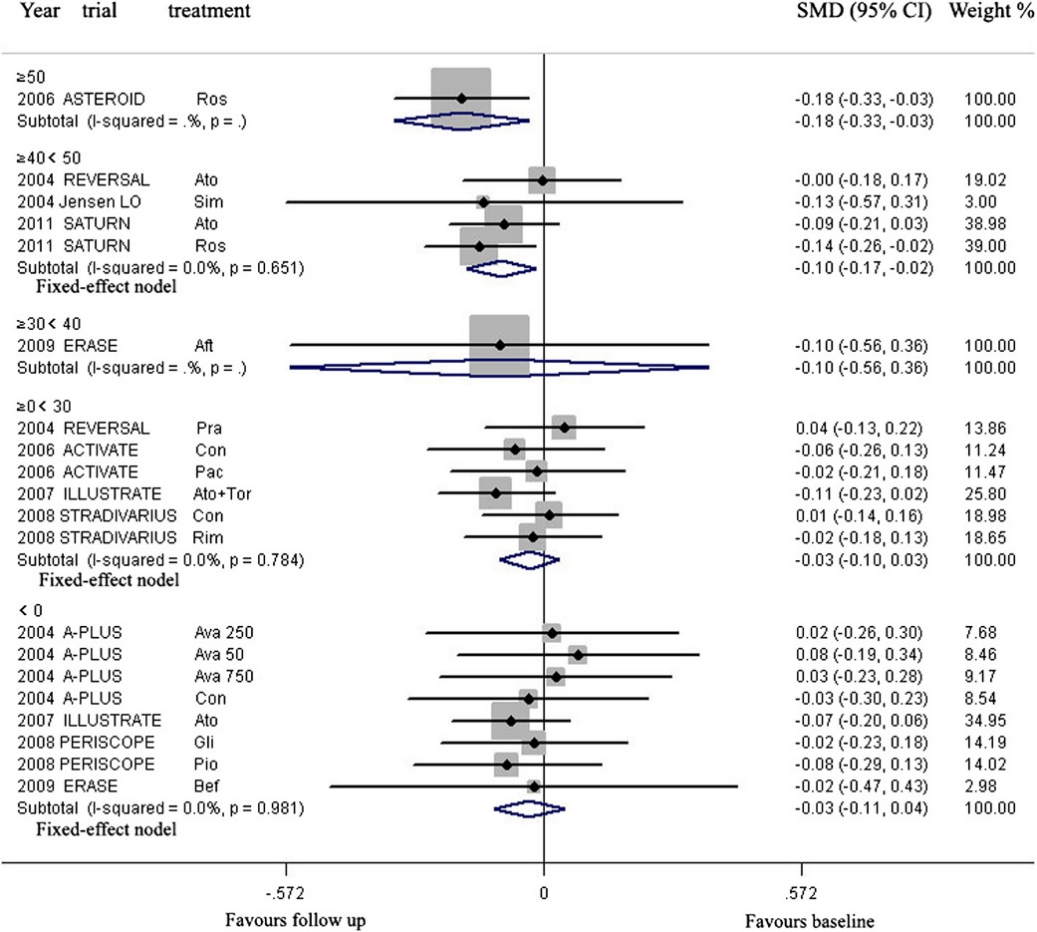
**Meta- analysis of the effects of reduction percentages of LDL-C at follow up on the regression of coronary atherosclerotic plaque in Western.** Abbreviation as in Figure 4.

**For Western**, meta-analysis showed that LDL-C lowering in group ≥40 < 50, ≥50% could lead to regression of CAP, but LDL-C lowering in group <0, ≥0 < 30% and ≥30 < 40 could not (Figure 6, Table 3).

In group ≥40 < 50% (including four arms) with mean 22.6 months of follow up, the volumes of CAP (143.1 mm^3^) at follow up were significantly decreased, compared with the volumes (148.8 mm^3^) at baseline (SMD −0.095 mm^3^, 95% CI −0.171 ~ −0.019, *p* = 0.014). There was no significant heterogeneity among arms (χ^*2*^ for heterogeneity = 1.64, *P* = 0.651, *I*^2^ = 0%).

Sensitivity analyses showed that LDL-C lowering in group ≥40 < 50 could still lead to regression of CAP with reduction of the plaque volume ranged from −0.065 mm^3^ (95% CI −0.163 ~ 0.032) when the arm of 2011 SATURN Ros was omitted to −0.116 mm^3^ (SMD, 95% CI −0.201 ~ −0.032) when 2004 REVERSAL Ato was omitted. Publication bias analysis suggested the values of *p* by Egger’s test were 0.804.

In group group <0, ≥0 < 30% and ≥30 < 40, meta-analysis were showed in Table 3.

**For Asian**, according to the reducing percentage of LDL-C at follow-up, the arms were grouped to following groups: ≥0 < 30, ≥30 < 40, ≥40 < 50.

**Figure 7:**
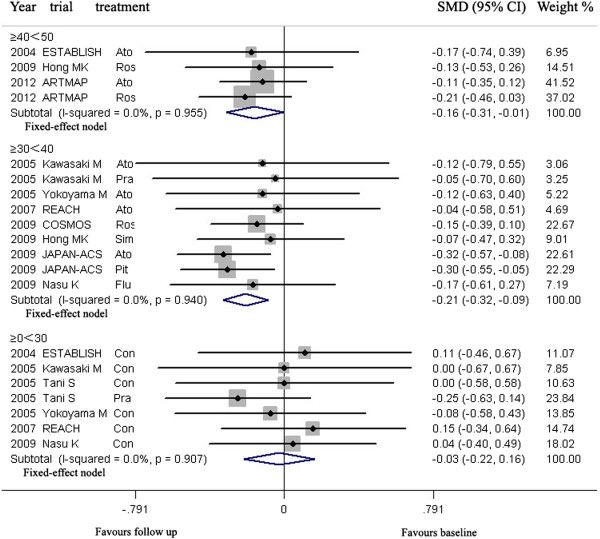
**Meta- analysis of the effects of reduction percentages of LDL-C at follow up on the regression of coronary atherosclerotic plaque in Asian.** Abbreviation as in Figure 4.

LDL-C lowering in group ≥30 < 40, ≥40 < 50% could lead to regression of CAP, but LDL-C lowering in group ≥0 < 30% could not (Figure 7, Table 3).

In group ≥30 < 40% (including nine arms) with mean 10.9 months of follow up, and group ≥40 < 50% (including four arms) with mean 6.9 months of follow up, the volumes of CAP (90.0, 179.9 mm^3^ respectively) at follow up were significantly decreased, compared with the volumes (98.6, 192.2 mm^3^ respectively) at baseline (SMD −0.206 mm^3^, 95% CI −0.324 ~ −0.088, *p* = 0.001; SMD −0.157 mm^3^, 95% CI −0.307 ~ −0.008, *p* = 0.039; respectively). There was no significant heterogeneity among arms (χ^*2*^ for heterogeneity = 2.91, *P* = 0.840, *I*^2^ = 0%; χ^*2*^ for heterogeneity = 0.33, *p* =0.955, *I*^2^ = 0%; for group ≥30 < 40, and group ≥40 < 50 respectively).

Sensitivity analyses showed that LDL-C lowering in group ≥30 < 40% could still lead to regression of CAP with reduction of the plaque volume ranged from −0.172 mm^3^ (95% CI −0.306 ~ −0.038) when the arm of 2009 JAPAN-ACS Ato was omitted to −0.223 mm^3^ (SMD, 95% CI −0.357 ~ −0.089) when 2009 COSMOS Ros was omitted. Publication bias analysis suggested that bias was significant with 0.004 of *p* value by Egger’s test.

Mean levels of LDL-C at baseline and follow up, mean reducing percentage of LDL-C in each group were showed in Table 4.

**Table 4:** **Levels and reducing percentage of LDL-C and duration in each group in Western and Asian (Mean ± SD)**

Group		N	Mean LDL-C at baseline (mg)	Mean LDL-C at follow up (mg)	Mean reducing percentage	Actual range of reducing percentage	Duration (month)
Western	≤70 mg	905	123.2 ± 6.9	61.9 ± 0.9	49.4 ± 3.5	37 ~ 53	23.1 ± 4.3
	>70 ≤ 100 HPmg	812	131.3 ± 15.2	73.6 ± 4.8	43.2 ± 2.2	41.5 ~ 46.7	21.7 ± 3.1
	>70 ≤ 100 MPmg	1548	91.3 ± 6.9	82.4 ± 8.2	9.0 ± 4.5	3.6 ~ 14.9	19.8 ± 2.7
	>70 ≤ 100 LPmg	1061	88.5 ± 5.5	91.5 ± 5.4	−4.7 ± 2.5	−1.7 ~ −8.5	19.9 ± 4.5
	>100 mg	464	123.4 ± 28.9	106.3 ± 4.4	8.7 ± 17.5	−10.9 ~ 25.0	18.0 ± 0.0
	>50%	349	130.4 ± 0.0	60.8 ± 0.0	53.4 ± 0.0	53.4 ~ 53.4	24.0 ± 0.0
	>40 ≤ 50%	1332	126.9 ± 13.1	69.3 ± 6.5	45.0 ± 2.8	41.5 ~ 47.8	22.6 ± 2.7
	>30 ≤ 40%	36	100.2 ± 30.2	63.1 ± 17.4	37.0	37 ~ 37	2.0 ± 0.0
	>0 ≤ 30%	1797	99.4 ± 21.4	86.2 ± 12.2	11.2 ± 6.9	3.6 ~ 25.0	19.5 ± 2.6
	<0%	1276	89.1 ± 5.3	93.2 ± 6.2	−5.6 ± 3.1	−1.7 ~ −10.9	19.6 ± 4.2
Asian	≤70 mg	345	111.5 ± 4.3	57.0 ± 5.0	47.2 ± 1.7	44 ~ 49	6.9 ± 2.1
	>70 ≤ 100 HPmg	540	134.2 ± 7.8	84.0 ± 5.0	36.1 ± 1.8	32.3 ~ 39.0	11.0 ± 2.2
	>100 mg	235	128.6 ± 10.5	117.2 ± 11.9	7.3 ± 10.7	0 ~ 32	7.8 ± 2.8
	>40 ≤ 50%	345	111.5 ± 4.3	57.0 ± 5.0	47.2 ± 1.7	44 ~ 49	6.9 ± 2.1
	>30 ≤ 40%	558	134.7 ± 8.1	84.6 ± 5.8	36.0 ± 1.9	32 ~ 39	10.9 ± 2.4
	>0 ≤ 30%	217	126.9 ± 9.1	118.3 ± 11.5	5.3 ± 8.3	0 ~ 20.0	8.0 ± 2.8

### The effect of lowering LDL-C by statins on regression of coronary atherosclerotic plaque in Western and Asian

**Figure 8:**
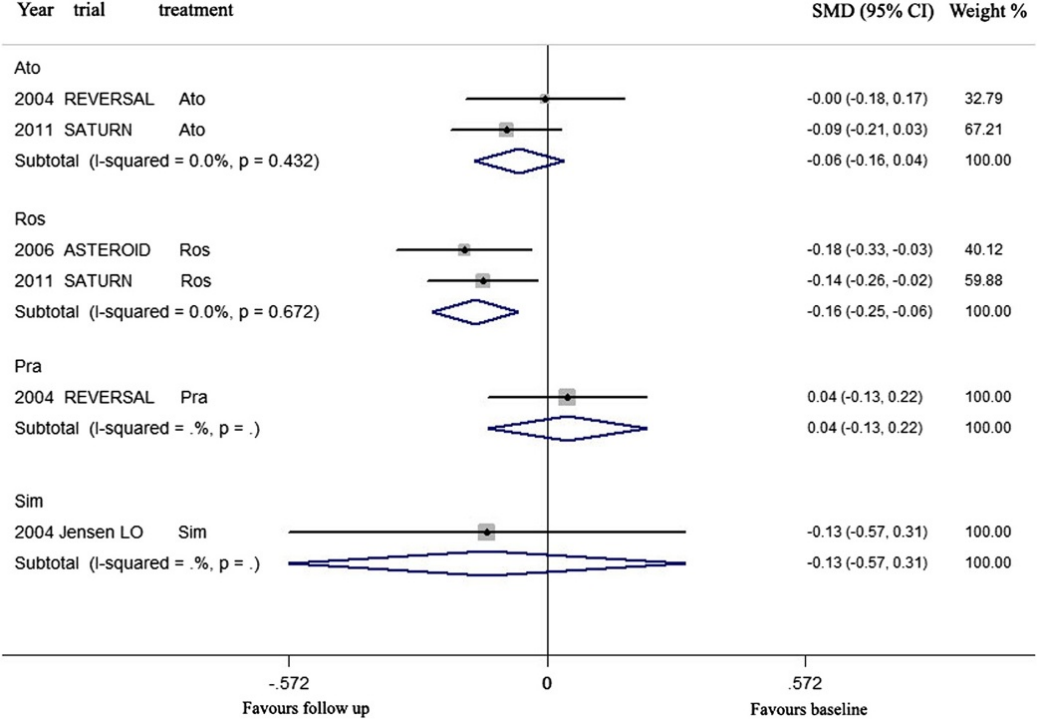
**Meta- analysis of the effects of LDL-C lowering by different statins on the regression of coronary atherosclerotic plaque in Western.** Abbreviation as in Figure 4.

**For Western**, atorvastatin, rosuvastatin, pravastatin and simvastatin were used in trials to investigate the effects of LDL-C lowering on CAP. Meta-analysis indicated that LDL-C lowering by rosuvastatin could lead to regression of CAP, but LDL-C lowering by atorvastatin, pravastatin, and simvastatin could not (Figure 8, Table 5).

**Table 5:** **Results of meta-analysis in different statins groups in Western and Asian**

Group		Included arms (and case)	Pooled SMD (95% CI, ***p*** )	Heterogeneity test		Sensitivity analyses		Egger’s test
				χ ^***2***^ test ( ***p*** )	***I*** ^***2***^	Lower SMD (95% CI)	Upper SMD (95% CI)	
Western	Rosuvastatin	2(869)	−0.158(−0.253 ~ −0.064, 0.001)	0.18(0.672)	0	−0.142 (−0.263 ~ −0.020) Without 2006 ASTEROID Ros	−0.183 (−0.332 ~ −0.035) Without 2011 SATURN Ros	0.000
	Atorvastatin	2(772)	−0.062(−0.162 ~ 0.038, 0.225)	0.62(0.432)	0			0.000
	Pravastatin	1(249)	0.045(−0.131 ~ 0.221, 0.616)					
	Simvastatin	1(40)	−0.133(−0.572 ~ 0.306, 0.552)					
Asian	Rosuvastatin	3(304)	−0.172(−0.331 ~ −0.012, 0.035)	0.17(0.917)	0	−0.143 (−0.352 ~ 0.066) Without 2012 ARTMAP Ros	−0.189 (−0.397 ~ 0.019) Without 2009 COSMOS Ros	0.660
	Atorvastatin	6(366)	−0.185(−0.330 ~ −0.040, 0.013)	1.94(0.858)	0	−0.113 (−0.292 ~ 0.068) Without 2009 JAPAN-ACS Ato	−0.230 (−0.417 ~ −0.044) Without 2012 ARTMAP Ato	0.456
	Pravastatin	2(70)	−0.197(−0.529 ~ 0.135, 0.245)	0.26(0.608)	0			
	Pitavastatin	1(125)	−0.304(−0.553 ~ −0.055, 0.017)					
	Fluvastatin	1(40)	−0.169(−0.608 ~ 0.270, 0.450)					
	Simvastatin	1(50)	−0.074(−0.467 ~ 0.318, 0.710)					

LDL-C lowering by rosuvastatin (mean 40.0 mg daily for mean 24 months) could significantly decrease the volumes of CAP at follow up, compared with the volumes at baseline (SMD −0.158 mm^3^, 95% CI: −0.253 ~ −0.064, *p* = 0.001). There was no significant heterogeneity among arms (χ^*2*^ for heterogeneity = 0.18, *p* =0.672, *I*^2^ = 0%).

Sensitivity analyses suggested that lowering LDL-C by rosuvastatin could lead to regression of CAP with reduction of the plaque volume ranged from −0.142 mm^3^ (SMD, 95% CI: −0.263 ~ −0.020) when the arm of 2006 ASTEROID Ros was omitted to −0.183 mm^3^ (SMD, 95% CI: −0.332 ~ −0.035) when the arm of 2011 SATURN Ros was omitted. But publication bias was found, the values of *p* by Egger’s test was 0.000 (Table 5).

**Figure 9:**
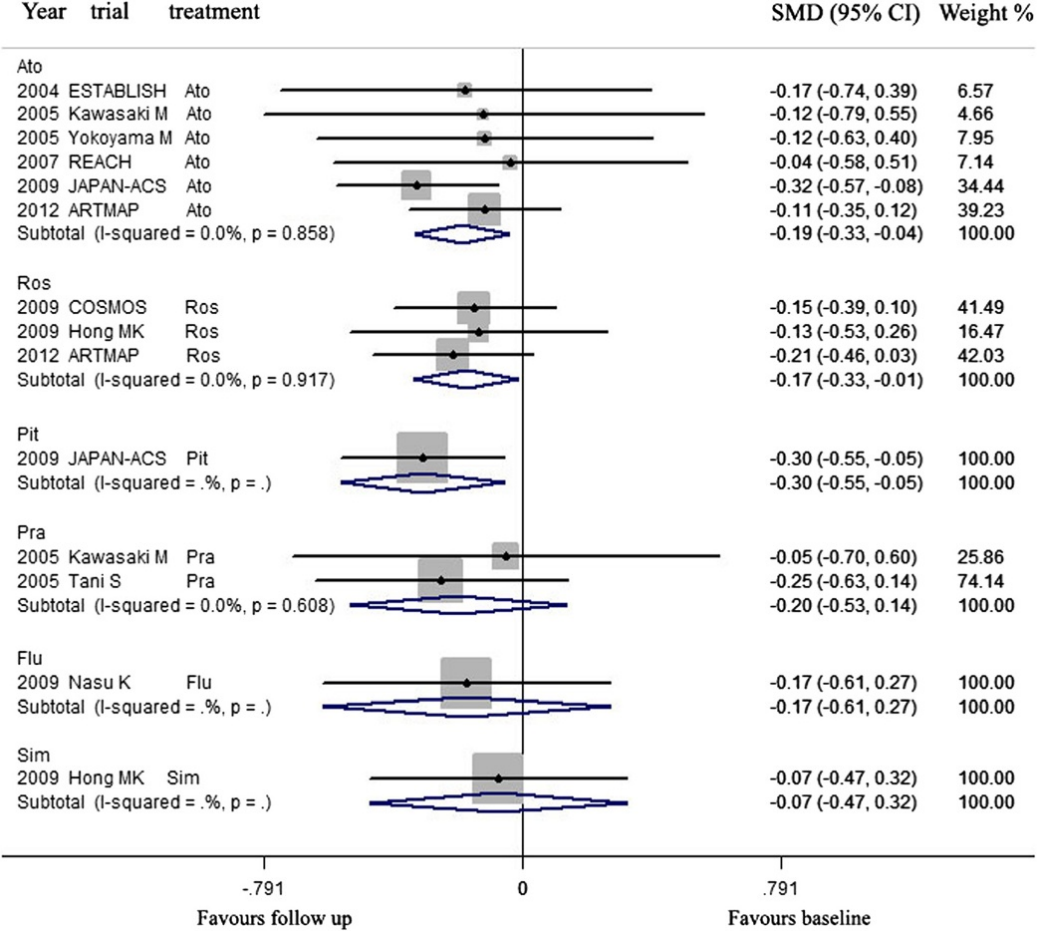
**Meta- analysis of the effects of LDL-C lowering by different statins on the regression of coronary atherosclerotic plaque in Asian.** Abbreviation as in Figure 4.

**For Asian**, atorvastatin, rosuvastatin, pitavastatin, pravastatin, fluvastatin and simvastatin were used in trials to investigate the effects of LDL-C lowering on CAP. Meta-analysis indicated that LDL-C lowering by rosuvastatin, atorvastatin could lead to regression of CAP, but LDL-C lowering by pitavastatin, pravastatin, fluvastatin and simvastatin could not (Figure 9, Table 5).

LDL-C lowering by rosuvastatin (mean 14.1 mg daily for mean 10.3 months), atorvastatin (mean 18.9 mg daily for mean 7.8 months) could significantly decrease the volumes of CAP at follow up, compared with the volumes at baseline (SMD −0.172 mm^3^, 95% CI: −0.331 ~ −0.012, *p* = 0.035; SMD −0.185, 95% CI: −0.330 ~ −0.040, *p* = 0.013; respectively). There was no significant heterogeneity among arms (χ^*2*^ for heterogeneity = 0.17, *p* =0.917, *I*^2^ = 0% for rosuvastatin; χ^*2*^ for heterogeneity = 1.94, *p* =0.858, *I*^2^ = 0% for atorvastatin).

Sensitivity analyses suggested that lowering LDL-C by rosuvastatin could not significantly lead to regression of CAP when the arm of 2012 ARTMAP Ros or 2009 COSMOS Ros was omitted. Also, Lowering LDL-C by atorvastatin could not significantly lead to regression of CAP when the arm of 2009 JAPAN-ACS Ato was omitted. No publication bias was found, the values of *p* by Egger’s test for rosuvastatin and atorvastatin group were 0.660, 0.456 respectively (Table 5).

Intensity of lowering LDL-C by different statins was shown in Table 6. Rosuvastatin and atorvastatin could reduce LDL-C by more than 40%.

**Table 6:** **Levels and reducing percentage of LDL-C, dosage and duration in different statin group in Western and Asian (Mean ± SD)**

Group		N	Age	MeanLDL-C at baseline (mg)	MeanLDL-C at follow up (mg)	Mean reducing percentage	Statin dosage (mg)	Duration (month)
Western	Rosuvastatin	869	57.8 ± 0.6	124.2 ± 5.1	61.9 ± 0.9	49.9 ± 2.6	40.0 ± 0.0	24.0 ± 0.0
	Atorvastatin	772	57.2 ± 1.0	129.8 ± 14.2	73.1 ± 4.1	43.0 ± 2.1	80.0 ± 0.0	22.0 ± 2.8
	Pravastatin	249	56.6 ± 0.0	150.2 ± 0.0	110.4 ± 0.0	25.0 ± 0.0	40.0 ± 0.0	18.0 ± 0.0
	Simvastatin	40	57.7 ± 0.0	158.7 ± 0.0	85.1 ± 0.0	46.7 ± 0.0	40.0 ± 0.0	25.0 ± 0.0
Asian	Rosuvastatin	304	58.9 ± 3.3	123.1 ± 14.6	67.2 ± 13.8	44.0 ± 4.8	14.1 ± 4.9	10.3 ± 3.7
	Atorvastatin	366	60.9 ± 3.0	124.1 ± 12.7	72.9 ± 14.2	40.7 ± 5.5	18.9 ± 2.9	7.8 ± 2.2
	Pitavastatin	125	62.5 ± 11.5	130.9 ± 33.3	81.1 ± 23.4	36.2 ± 19.5	4	8 ~ 12
	Pravastatin	70	64.0 ± 1.8	134.9 ± 8.4	130.5 ± 0.9	23.1 ± 5.3	16.3 ± 2.2	6.0 ± 0.0
	Fluvastatin	40	63.0 ± 10.0	144.9 ± 31.5	98.1 ± 12.7	32.3	60	12
	Simvastatin	50	58.0 ± 0.0	119.0 ± 0.0	78.0 ± 0.0	34.5 ± 0.0	20.0 ± 0.0	12.0 ± 0.0

### The difference between Western and Asian in usage of statins

The meta analysis showed that rosuvastatin and atorvastatin can regress CAP (Table 5). LDL-C levels, intensity of lowering LDL-C by rosuvastatin and atorvastatin, its dosage and duration were compared between Western and Asian (Table 7). Intensity of lowering LDL-C by rosuvastatin and atorvastatin in Western group were similar to that in Asian group, but the dosages of rosuvastatin and atorvastatin in Asian group were significantly lower than those in Western group, and the duration of statins administration in Asian group were significantly shorter than those in Western, as showed in Table 7.

**Table 7:** **Comparison between Western and Asian in rosuvastatin and atorvastatin**

	Rosuvastatin			Atorvastatin		
	Western	Asian	p	Western	Asian	p
N/arm	869/2	304/3		772/2	366/6	
Mean LDL-C at baseline (mg)	124.2 ± 5.1	123.1 ± 14.6	0.928	129.8 ± 14.2	124.1 ± 12.7	0.610
Mean LDL-C at follow up (mg)	61.9 ± 0.9	67.2 ± 13.8	0.642	73.1 ± 4.1	72.9 ± 14.2	0.986
LDL-C Mean reducing percentage	49.9 ± 2.6	44.0 ± 4.8	0.221	43.0 ± 2.1	40.7 ± 5.5	0.600
Statin dosage (mg)	40.0 ± 0.0	14.1 ± 4.9	0.006	80.0 ± 0.0	18.9 ± 2.9	<0.001
Duration (month)	24.0 ± 0.0	10.3 ± 3.7	0.016	22.0 ± 2.8	7.8 ± 2.2	<0.001

## Discussion

This meta-analysis revealed that intensive LDL-C lowering can regress CAP both in Western and Asian. For regressing CAP, the dosage of statins administrated in Westerns was different from that in Asians. Asians need lower dosage of atorvastatin or rosuvastatin than Westerns though there was no difference between Westerns and Asians in pharmacokinetic and pharmacodynamic study [32, 33].

### The effect difference of LDL-C lowering on CAP between Western and Asian

For Western including American, Canadian, German, French, English, Australian and Dane [10, 5–7, 16, 23–26, 28], the meta-analysis (Table 3) in subgroup ≤70 mg and ≥40 < 50% of Western indicated that LDL-C level lowering to <69.3 mg or reducing by > 45% for 22.6 months of follow up (Table 4) could lead to regression of CAP, but the meta-analysis (Table 3) in subgroup >70 ≤ 100 HP mg of Western showed that LDL-C level lowering to 73.2 mg or reducing by 43.6% for 21.7 months of follow up (Table 4) was not enough for regressing CAP.

For Asian including Japanese and Korean [20, 11–15, 17, 22, 29, 30], the meta-analysis in subgroup ≤70 mg and ≥40 < 50% of Asian indicated that LDL-C level lowering to 57.0 mg or reducing by 47.2% for 6.9 months of follow up could lead to regression of CAP, but sensitivity analyses showed that LDL-C lowering in this two subgroup could not significantly lead to regression of CAP when the arm of 2012 ARTMAP Ros or 2012 ARTMAP Ato was omitted (Table 3). The meta-analysis in subgroup ≥ 30 < 40% of Asian indicated that LDL-C level lowering to 84.6 mg or reducing by 36.0% for 10.9 months of follow up could also lead to regression of CAP, but publication bias was significant. The meta-analysis in subgroup >70 ≤ 100HP mg of Asian with good sensitivity and no publication bias indicated that LDL-C level lowering to 84.0 mg or reducing by 36.1% for 11 months of follow up with could lead to regression of CAP (Table 3).

Taken all the results of meta-analysis together, for Western, it was recommended that LDL-C level might be reduced by >45% or to a target level < 69 mg/dL for regressing CAP; for Asian, LDL-C level might be reduced by >36% or to a target level < 84 mg/dL.

### Different effects of statins on Westerns and Asians

Whether statins has different effect on Westerns and Asians remains to be settled.

The study by Lee E et al. [34] and MEGA Study [35] suggested statins have different effects on Westerns and Asians. In 2005, Lee E et al. [34] prospectively examined the pharmacokinetics of rosuvastatin in White and Asian individuals living in Singapore, and reported that plasma exposure to rosuvastatin and its metabolites was significantly higher in Chinese, Malay, and Asian-Indian subjects compared with Western subjects living in the same environment. But the mechanisms underlying ethnic differences in rosuvastatin disposition remain to be unearthed [36]. MEGA Study [35] indicated that a small dose of pravastatin that was half the dose administered to western patients, reduced LDL-C by 19-22% (which is lower than that reductions of 23–35% in western patients), but could substantially reduce the risk of coronary heart disease in Japanese.

But two meta-analysis did not demonstrate the difference of rosuvastatin and atorvastatin on Westerns and Asians. The meta-analysis including the 36 trials of pharmacodynamics of rosuvastatin in Western and Asian hypercholesterolemia patients did not confirm that there was significant difference in the exposure-response relationship for LDL-C reduction between Westerners and Asians [33].The meta-analysis including 22 pharmacokinetic studies also demonstrated no differences in the systemic exposure to atorvastatin between Asian and Caucasian subjects [32].

Our meta-analysis revealed that there were difference of rosuvastatin and atorvastatin in lowering LDL-C and regressing CAP between Westerns and Asians. The meta-analysis of rosuvastatin including 2 trials with 869 Western patients indicated that 40 mg of rosuvastatin daily for 24 months with reducing LDL-C by 49.9% could regress CAP. But the meta-analysis of rosuvastatin including 3 trials with 304 Asian patients showed that 14.1 mg of rosuvastatin daily for 10.3 months with reducing LDL-C by 44.0% could also regress CAP though the result of sensitivity analyses is not as good as that in Western (Table 5). The meta-analysis of atorvastatin including 2 trials with 772 Western patients showed that 80 mg of atorvastatin daily for 22 months with reducing LDL-C by 43.0% could not significantly regress CAP. But the meta-analysis of atorvastatin including 6 trials with 366 Asian patients demonstrated that 18.9 mg of atorvastatin daily for 7.8 months with reducing LDL-C by 40.7% could significantly regress CAP though the result of sensitivity analyses is not as good as that expected (Table 5).

Comparison between Western and Asian in using rosuvastatin and atorvastatin indicated that the dosages of rosuvastatin and atorvastatin in Asian group were significantly lower than those in Western (Table 7).

Based on this meta-analysis, reducing LDL-C by >40% in Westerns need atorvastatin 80 mg or rosuvastatin 40 mg, but in Asians need only atorvastatin 18.9 mg or rosuvastatin 14.1 mg. For regressing CAP, 40 mg of rosuvastatin might be daily administrated in Western for 24 months; 14.1 mg of rosuvastatin or 18.9 mg of atorvastatin might be daily administrated in Asian for 10.7 or 7.8 months respectively.

### Study limitation

As with the meta-analysis [3], this study has some limitations. There might be publication bias, difference of the method detected and follow up duration. But those differences in measurements and plaque selection did not affect the change of the target plaque with LDL-C levels. So, it has little effect on homogeneous of studies, and on the relationship between CAP change and LDL-C level. But the trials of single statin on LDL-C and CAP of specific population (for example, 2 trials about atorvastatin on Western with 727 participants or 6 on Asian with 366 in Table 5) were limited, the effect of statin on specific population remains to be investigated. The duration of follow up between Western and Asian was different (Table 4, 6 and 7), and treatment duration might have some effect on CAP regression. But the trials from Asian and Western were respectively meta-analysed in this study. Therefore, the difference in follow-up duration between Asian and Western did not influence the results of the meta-analysis. The CAP regression in short period of statins therapy in Asian suggested that the CAPs in Asian were easily regressed by statins.

This meta-analysis did not investigate the effect of reduction of LDL-C on adverse cardiovascular events because all participants of the included trial must be alive at follow up. But in the Extended-ESTABLISH study, the incidence of adverse cardiovascular events in statin group with CAP regression were reduced to half that seen in the control group [37]. In the Extended JAPAN-ACS study [38], there was no significantly different association of incidence of adverse cardiovascular events with the CAP regression extent, but that greater external elastic membrane volume regression (<−6.56%) had a significantly lower incidence of cumulative events than the lesser suggested the importance of CAP regression in reducing adverse cardiovascular events. A meta-analysis [39] included 7864 CAD patients showed that rates of plaque volume regression were significantly associated with the incidence of MI or revascularization.

## Conclusions

LDL-C lowering therapy has a different effect on atherosclerotic plaque between Westerns and Asians. This systemic review demonstrated that there is a different effect of LDL-C lowering on CAP between Westerns and Asians. For regressing CAP, Asians need lower dosage of statins or lower intensity LDL-C lowering therapy (by >36%) than Westerns (by 45%).

## Abbreviations

ACAT: Acyl–coenzyme A: cholesterol acyltransferase
ACS: Acute coronary syndrome
ATP III: Adult Treatment Panel III
CAD: Coronary artery disease
CAG: Coronary angiography
CAP: Coronary atherosclerotic plaque
CETP: Cholesteryl ester transfer protein
CHD: Coronary heart disease
IVUS: Intravascular ultrasound
CI: Confidence interval
LDL-C: Low-density lipoprotein cholesterol
RCT: Randomized controlled trial
SMD: Standardized mean differences.
